# The Onset of Systemic Oxidative Stress Associated with the Accumulation of Lipid Peroxidation Product Acrolein in the Skin of Patients with Small-Vessel Vasculitis

**DOI:** 10.3390/molecules26082344

**Published:** 2021-04-17

**Authors:** Vesna Sredoja Tisma, Stela Bulimbasic, Danica Galesic Ljubanovic, Kresimir Galesic, Jadranka Morovic-Vergles, Josko Mitrovic, Koji Uchida, Franz Tatzber, Neven Zarkovic, Morana Jaganjac

**Affiliations:** 1 Polyclinic Department of Dermatology and Venereology, Dubrava University Hospital, 10040 Zagreb, Croatia; vtisma@kbd.hr; 2 Department of Pathology and Cytology, University Hospital Centre Zagreb, 10000 Zagreb, Croatia; stela.bulimbasic@gmail.com; 3 Department of Pathology, School of Medicine Zagreb, University of Zagreb, 10000 Zagreb, Croatia; danica.ljubanovic@mef.hr; 4 Department of Nephropathology and Electron Microscopy, Dubrava University Hospital, 10040 Zagreb, Croatia; 5 Division of Nephrology, Department of Internal Medicine, Dubrava University Hospital, University of Zagreb School of Medicine, 10040 Zagreb, Croatia; kresog@kbd.hr; 6 Division of Clinical Immunology, Allergology and Rheumatology, Department of Internal Medicine, Dubrava University Hospital, University of Zagreb School of Medicine, 10040 Zagreb, Croatia; jmorovic@kbd.hr (J.M.-V.); jmitrovi@kbd.hr (J.M.); 7 Laboratory of Food and Biodynamics, Graduate School of Bioagricultural Sciences, Nagoya University, Nagoya 464-8601, Japan; a-uchida@g.ecc.u-tokyo.ac.jp; 8 Omnignostica Gmbh & Co. KG, 2465 Höflein, Austria; franz@tatzber.at; 9 Laboratory for Oxidative Stress, Division of Molecular Medicine Rudjer Boskovic Institute, 10000 Zagreb, Croatia; zarkovic@irb.hr

**Keywords:** vasculitis, oxidative stress, lipid peroxidation, acrolein

## Abstract

Small-vessel vasculitis (SVV) is the inflammation of the vessel wall that can result in hemorrhage and/or ischemia. Among the histological findings in SVV are increased infiltrating neutrophils, which, due to their oxidative burst and myeloperoxidase activity, release excessive reactive oxygen species, triggering a chain reaction of lipid peroxidation and yielding reactive aldehydes such as acrolein. The implication of oxidative stress in the pathogenesis of SVV was studied, focusing on acrolein immunohistochemistry in the affected skin vessels and systemic stress response. Samples from SVV patients and healthy subjects were collected and analyzed for total serum peroxides, total antioxidant capacity, inflammatory and immunological parameters, as well as for the presence of acrolein–protein adducts in the skin tissue specimens. The obtained data showed that systemic redox homeostasis and iron metabolism are altered in SVV patients. Possible biomarkers in the evaluation of oxidative status, disease activity and prevalence were indicated. Furthermore, a strong correlation between the accumulation of acrolein–protein adducts in the skin and the progression of the disease was revealed. Thus, the results of this study demonstrate that SVV is not only associated with systemic oxidative stress but also with tissue-specific oxidative stress that promotes acrolein formation and protein modification correlating with the severity of cutaneous vasculitis.

## 1. Introduction

Vasculitis is the inflammation of blood vessels that can cause damage to vessel walls, leading to hemorrhage and/or ischemia [1,2]. Small-vessel vasculitis (SVV) is a vasculitis that affects small intraparenchymal arteries, arterioles, capillaries, venules and veins [2]. According to the 2012 Revisited International Chapel Hill Consensus Conference Nomenclature of Vasculitides, cutaneous vasculitis is included in both SVV and single-organ vasculitis groups of nomenclature [2]. The skin, as the largest human organ with a large vascular bed, is often involved in many vasculitis syndromes [3]. Cutaneous vasculitis can be either a primary (idiopathic) disorder or a secondary disorder resulting from drug administration such as beta-lactam antibiotics, thiazide diuretics or nonsteroidal anti-inflammatory drugs. Additionally, food or food additives, infection or diseases such as connective tissue disease, inflammatory bowel disease, paraproteinemia and malignancies may trigger the development of cutaneous vasculitis [3,4].

Vasculitis may present clinically as a localized cutaneous disease with a benign course, which sometimes can rapidly, concomitantly or sequentially progress into systemic disease. The most commonly affected internal organs in systemic vasculitis are kidneys, joints, the gastrointestinal tract, the lungs, the nervous system, the musculoskeletal system and the eyes. Diagnostic evaluation of patients with cutaneous vasculitis depends on clinical signs and symptoms. The course of cutaneous vasculitis may be acute, recurrent or chronic. Early diagnosis of SVV and the recognition of systemic involvement, followed by appropriate therapy, is required in order to restore or preserve the function of vital organs [1,3,4,5].

Although the underlying mechanisms of different forms of SVV are not completely understood, inflammation of blood vessel walls is a common defining feature and the central process of all categories of vasculitis [2]. Oxidative stress, redox signaling and the resulting lipid peroxidation are involved in various and numerous pathological states, including inflammation [6,7]. In addition, active vasculitis is often accompanied by anemia [8], marked by decreased serum iron and elevated serum ferritin [9].

The most common histological finding in SVV is leukocytoclastic angiitis involving dermal postcapillary venules [10], where neutrophils release powerful inflammatory mediators. A respiratory burst of activated neutrophils generates excessive reactive oxygen species (ROS) that can damage macromolecules [11,12]. Depending on the type and diffusion distance, ROS can induce oxidative damage to nucleic acids, proteins and lipids [12,13,14]. Peroxidation of polyunsaturated fatty acids (PUFAs) can trigger a chain reaction of lipid peroxidation, altering membrane fluidity and the function and yielding the formation of reactive aldehydes, such as acrolein [11,15,16].

In the body, acrolein can also be formed during the catabolism of polyamines and amino acids or as a metabolite of some drugs, such as cyclophosphamide [17,18]. Due to its electrophilic properties, acrolein can effectively bind and form adducts with DNA and proteins, altering their structure and function [17,19,20]. The evidence suggests that acrolein induces endothelial cell damage in vitro [21].

In the present work, we investigated the association of inflammation, inflammatory parameters, iron metabolism and systemic redox homeostasis with the pathology of SVV and, for the first time, the involvement of acrolein in the pathology of SVV disease.

## 2. Results

The histopathological analysis of 97 samples confirmed SVV in 67 patients, while 30 samples were normal skin. General characteristics of subjects whose biopsy samples were analyzed for the presence of acrolein–protein conjugates are summarized in Table 1.

The most common finding in the SVV patients was leukocytoclastic vasculitis. At the time of diagnosis, 16.7% of patients were in the florid phase of the disease, 66.6% were in the active phase of the disease and 16.7% were in the regression period. The average age of all subjects was 56.4 years, ranging from 19 to 88 years.

**Figure 1:**
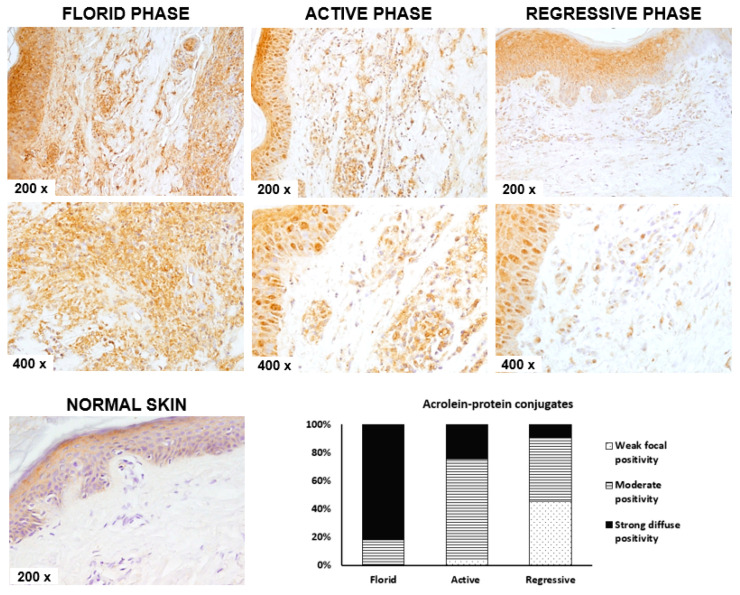
Immunohistochemical appearance of acrolein–protein conjugates in normal-appearing skin without small-vessel vasculitis (SVV) (*n* = 30) and the skin of a patient in the regression period of SVV (*n* = 11), in the active phase of SVV (m = 45) and in the florid phase of SVV (*n* = 11). Representative images at 200× and 400× magnifications are shown. The bar chart shows the semiquantitative analysis of the incidence of acrolein–protein conjugates in the skin with different phases of SVV.

The immunohistochemical appearance of acrolein–protein conjugates in the endothelium and walls of small blood vessels and the surrounding skin is shown in Figure 1. Acrolein–protein conjugates were not detected in the blood vessels and surrounding skin in the clinically healthy skin. On the contrary, all skin samples of SVV patients were positive for acrolein but contained variable amounts depending on the phase of the disease (Chi-square: 29.592, *p* < 0.001).

The skin samples with the mildest form of the disease, i.e., vasculitis regression phase, contained a low number of cells showing weak positivity to acrolein. A larger number of cells with moderate acrolein positivity was detected in skin samples from patients with an active SVV phase, while the highest level of acrolein–protein conjugates was detected in the skin samples of patients with the most severe SVV, the florid phase.

The presence of acrolein–protein conjugates depended on disease activity. Out of 11 patients with florid-phase vasculitis, most of the skin samples showed strong diffuse positivity (81.8%), while the remaining samples belonging to the same group had moderate positivity. Furthermore, most of the skin samples from the active SVV phase expressed moderate acrolein positivity (71.1%), followed by 24.4% of samples with strong diffuse positivity. Weak focal positivity was detected only in 4.4% of samples of the acute SVV phase. Finally, in the mildest form of the disease, the regression phase of SVV, most of the samples showed weak focal and moderate positivity (45.5% each), while only one sample expressed strong diffuse positivity (9%).

Further systemic effects of SVV were studied in the serum samples collected prospectively. Characteristics of subjects from the prospective cohort are summarized in Table 2. 

The mean age at diagnosis was 56.63 ± 16.85 years, ranging from 19 to 79 years. The mean duration of the disease was 8.7 ± 15.6 months, ranging from 1 day to 6 years. Most of the patients recruited showed associated symptoms and signs of systemic vasculitis (90%) and had associated chronic disease (80%). One patient had an associated malignancy, histopathologically verified as hepatocellular carcinoma, as part of an associated chronic hepatitis C virus infection, and one patient showed a habit of regular alcohol consumption in the anamnestic data. In addition, seven patients were smokers, while one patient died during the follow-up of the disease due to complications of systemic vasculitis. The control group consisted of healthy subjects with clinically healthy skin who do not smoke, do not consume alcohol or take any medications and who, by age and gender, were comparable to the prospective group of patients with vasculitis. 

The most common associated systemic symptom in prospective patients with SVV is fever (50%), followed by arthralgias and joint swelling (36.7% for both). One-third of prospective patients had anamnestic symptoms of cough, and the presence of blood in the urine was reported by 30% of SVV patients. Among the less common systemic symptoms present in less than 25% of prospective patients were headaches, myalgias, abdominal pain, diarrhea and paresthesia. 

A comparative analysis of all inflammatory and biochemical parameters measured in the prospective cohort, both control subjects and SVV patients, is shown in Supplementary Table S1. 

The obtained results show that patients with SVV have significantly elevated C-reactive protein (CRP), leukocytes (LEU), neutrophils (NEUT), monocytes (MONO), platelets (Trc), ferritin, gamma-glutamyl transferase (GGT), lactate dehydrogenase (LDH), urea, creatinine (CREAT) and phosphates (PHOS). On the contrary, creatine kinase (CK), hemoglobin (Hgb), hematocrit (Htc), iron (Fe) and total iron-binding capacity (TIBC) values were significantly reduced in SVV patients compared to healthy controls.

**Figure 2:**
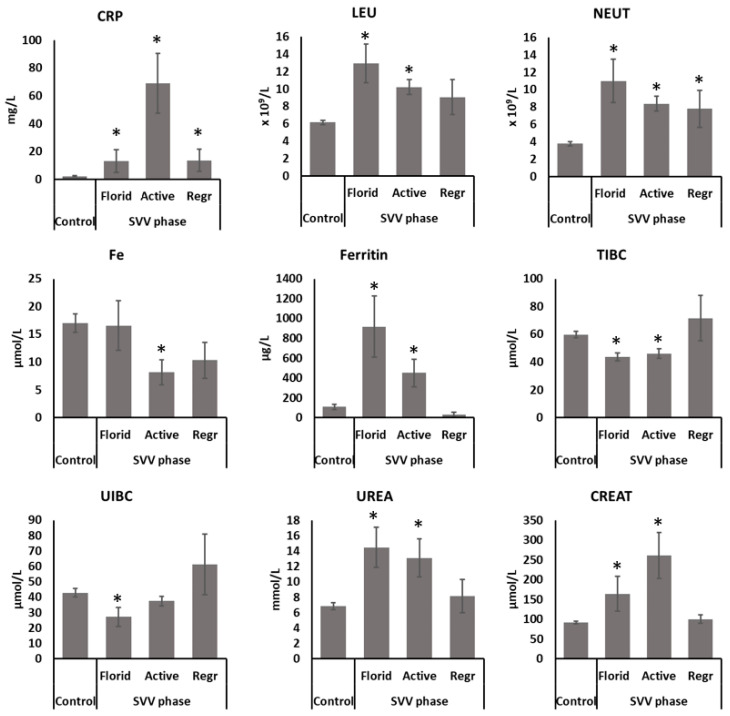
Parameters associated with the development of the disease. Significance * *p* < 0.05 compared to healthy controls.

Further analysis of changes in various parameters highlighted those associated with the development of the disease (Figure 2). Systemic inflammatory parameters CRP, leukocyte count and neutrophil count were significantly increased in the SVV patients (*p* < 0.05 for all). Furthermore, CRP values were dependent on disease activity, with the highest being in the active phase of the disease (*p* < 0.05 compared to the control group), while the others were only slightly increased in the serum samples of patients with a florid or regression phase (*p* < 0.05 for both). Both leukocyte and neutrophil count were the highest in the florid phase of the disease, while the values decreased in the active and regression phase of the disease, although still significantly elevated compared to the controls (*p* < 0.05 for all). 

Moreover, Fe, ferritin, TIBC and unsaturated iron-binding capacity (UIBC), biochemical parameters of iron metabolism, were altered in the SVV patients. Although the patients in the florid phase had significantly elevated ferritin, Fe level was comparable to the control, while UIBC and TIBC were significantly decreased (*p* > 0.05). In the active phase, Fe level, together with TIBC and UIBC, was decreased compared to the healthy control. Ferritin level was lower in the active phase than in the florid phase but still elevated compared to the control. With the disease regression, all four parameters returned to normal values and did not significantly differ compared to healthy controls. 

Finally, urea and creatinine biochemical parameters were elevated in SVV patients, indicating renal dysfunction. Urea and creatinine levels also depended on disease activity, with urea being the highest in the florid phase (*p* < 0.05), while creatinine was the highest in the active phase of the disease. With disease regression, both urea and creatinine values returned to control values (*p* > 0.05).

**Figure 3:**
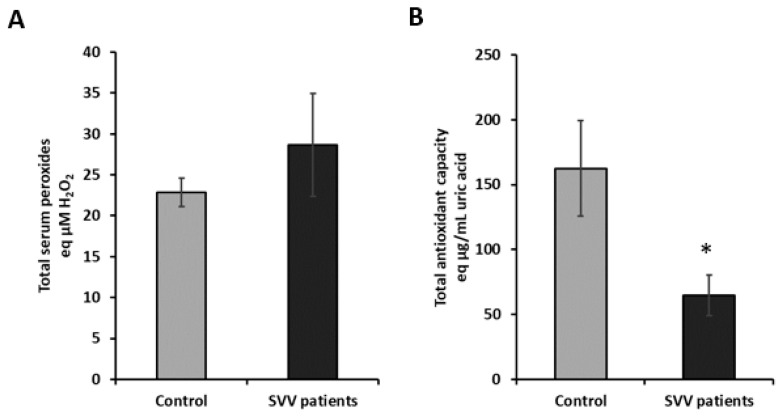
Body redox homeostasis is altered in SVV patients. (**A**) Total serum peroxides; (**B**) total antioxidative capacity. Significance * *p* < 0.05 compared to control.

The analysis of body redox homeostasis points to the involvement of oxidative stress in SVV (Figure 3). The level of serum peroxides was almost 20% higher in patients with SVV than in healthy control subjects; however, it did not reach significance due to the sample size (*p* > 0.05, Figure 3A). On the contrary, total antioxidant capacity was significantly decreased in SVV patients compared to healthy control samples (*p* ˂ 0.05, Figure 3B). Antioxidant capacity was decreased by more than 35% lower in SVV patients.

The correlation analyses revealed a significant positive correlation between serum peroxides with the CRP (Pearson correlation: 0.584, *p* = 0.009).

## 3. Discussion

Although most of the patients with skin SVV present mild, self-limited conditions, they could be the first sign of more serious systemic disease with multiorgan involvement. It is critically important to distinguish skin-limited presentations of SVV from severe life-threatening systemic SVV as an initial manifestation. It can be challenging to determine which patients presenting with cutaneous SVV are at risk of systemic disease; therefore, identifying predictive markers of the presence of systemic disease is very important. All patients included in this study had cutaneous manifestations of SVV, and the most common histological finding was leukocytoclastic vasculitis, with fever being the most common systemic symptom. These results are in agreement with the earlier study conducted in 160 patients with leukocytoclastic vasculitis that showed that paresthesia, fever and the absence of painful lesions are prognostic risk factors for systemic involvement [22]. As there is no ideal classification of SVV, in this study, patients were grouped based on the pathohistological findings of the biopsy of vasculitic skin changes: (1) patients in the florid phase of the disease; (2) patients in the active phase of the disease; and (3) patients in the regressive phase of the disease. The obtained distribution of the subjects, according to the phase of the disease, age and gender, is in agreement with the data from the literature [1,23]. The SVV was most common in adults with similar incidence in females and males in all three phases of the disease. 

The distinct mechanisms that could either lead to the damage of blood vessels in leukocytoclastic vasculitis or to inflammatory conditions are still controversial [24]. One of the mechanisms that could lead to blood vessel damage is oxidative stress. Guilpain and colleagues [25] reported that antimyeloperoxidase antibodies could play a pathogenic role in vivo by triggering an oxidative burst, leading to severe endothelial damage. A better understanding of the involvement of oxidative stress could help to develop more specific treatments for SVV rather than the relatively nonspecific anti-inflammatory and immunosuppressive approaches that are used [26]. 

It should also be mentioned that aging of the skin itself, especially in the case of photoaging, is associated with chronic inflammatory processes, oxidative stress and lipid peroxidation. Thus, generated reactive aldehyde 4-hydrohynonenal (4-HNE) was found to play an important role in the pathogenesis of skin aging [27]. However, the 4-HNE-induced alteration of skin elastin production associated with the pathophysiology of skin aging appeared to be different from the age-associated changes in the case of atherosclerosis, which is also considered chronic inflammatory vascular disease [28]. Hence, differences between the systemic appearance of lipid peroxidation products in different organs indicate different pathophysiological mechanisms of lipid peroxidation involved. If so, in the case of major blood vessels or internal organs, this could be mostly due to metabolic alterations (accumulation of oxidized lipids), while in the case of small vessels, inflammatory processes could be crucial, especially in the case of skin exposed to various stressors.

The accumulation of neutrophils in the SVV tissue could contribute to the disbalance in redox homeostasis by triggering the production of ROS [11]. Although at low concentrations, ROS may have an important signaling role, at higher concentrations, ROS are toxic [6,12]. Neutrophil-derived ROS can trigger a chain reaction of lipid peroxidation in skin tissues, yielding the formation of reactive aldehydes such as acrolein [11]. Moreover, the local cutaneous infiltration of leukocytes followed by their activation will also result in the release of myeloperoxidase that can further contribute to the local accumulation of acrolein via myeloperoxidase–hydrogen peroxide–chloride systems. Indeed, immunohistochemical analysis of skin biopsies for the presence of acrolein–protein adducts revealed that the intensity of acrolein–protein adducts in the skin of patients with SVV is proportional to the severity of skin disease.

However, excessive ROS will trigger lipid peroxidation, leading to the formation of reactive aldehydes, such as acrolein.

The results of the analysis of biochemical and inflammatory parameters suggest the monitoring of CRP, LEU, NEUT, Fe, ferritin, urea and creatinine parameters as possible biomarkers in the evaluation of patient oxidative status, disease activity and prevalence. Though the CRP levels were significantly elevated in patients’ samples of all phases of SVV, the highest CRP values were recorded in the group of patients in the active phase of the disease. CRP, a protein of the acute phase of inflammation, has a role in promoting the local proinflammatory effect. Therefore, elevated CRP may affect the course of the disease by inducing local complement activation and consequently enhancing local inflammation and cell damage [29,30]. Although CRP is used to monitor the SVV activity and can indicate a relapse of the disease, an elevation in CRP can also be induced by other events. Similarly, the progression of localized forms of SVV might not trigger the rise in CRP. This, together with the small sample size, could explain the discrepancies observed in the CRP levels in the florid and active phases of SVV. SVV patients also had pronounced leukocytosis and neutrophilia in the blood samples, which is complementary to the histological finding of neutrophilic and leukocytoclastic vasculitis in skin biopsies. Patients with a florid phase of the disease had the highest number of inflammatory cells in circulation, whose activation and release of inflammatory mediators, including ROS, probably contributed to the development of SVV.

Moreover, altered ferritin, Fe, TIBC and UIBC in SVV patients indicate impaired iron metabolism. The involvement of oxidative stress and altered iron metabolism was earlier reported for another skin disease, rosacea [7]. Ferritin is a protein that can quickly store and also release iron. In the inflammatory reaction in SVV, the superoxide anion produced by neutrophils may induce iron mobilization [31]. Elevated iron promotes ROS formation [32], among which of particular relevance is a highly reactive hydroxyl radical that can trigger a chain reaction of lipid peroxidation and may also induce ferroptotic cell death [33].

Additionally, elevated urea and creatinine values in SVV patients confirm earlier studies [34], indicating that the kidneys are among the most frequently affected organs. Thus, early detection and the timely treatment of renal dysfunction in SVV patients are extremely important to avoid later serious complications such as renal failure. We further detected elevated levels of serum peroxides in SVV patients, while the antioxidant capacity was decreased, pointing to altered body redox homeostasis.

In conclusion, the observed differences between patients and healthy subjects suggest the involvement of oxidative stress, increased lipid peroxidation and imbalance in the antioxidant defense system in the pathogenesis of the SVV disease. Therefore, the suppression of oxidative stress might be a potentially useful strategy for the treatment of SVV. This study may also support the search for novel pharmacologic agents with antioxidant properties to treat affected patients. We also suggest that impaired oxidant/antioxidant balance should be taken into consideration in the follow-up of patients with SVV. The serum markers and immunohistochemical assessment of acrolein content in the skin of patients with SVV could help in assessing the activity of SVV and selecting the most appropriate treatments. Therefore, further studies with a higher number of SVV patients and long-term monitoring are desirable to evaluate in more detail the potential causative role of oxidative stress and acrolein in SVV. Because the pathogenesis of inflammatory and stress-associated diseases is complex, especially in the case of a systemic stress response, it could hardly be expected to identify a single biomarker for monitoring the diversity and the multisystem nature of vasculitis. However, the results of this study, for the first time, impose the important role of acrolein in the assessment of disease activity.

## 4. Materials and Methods

### 4.1. Samples, Data Collection and Protection of Human Participants

Data and skin samples from 30 healthy controls and 37 histologically confirmed SVV patients were collected and evaluated retrospectively from the existing database of the Department of Pathology and Cytology, University Hospital Dubrava, Zagreb, Croatia. 

Prospectively, skin and serum samples from 30 SVV patients were collected in the period between 2011 and 2012. In addition, 30 serum samples from control healthy subjects, matched for age and gender, were collected on the same day in the period between 2011 and 2012. Neither patients nor control subjects had a history of any topical or systemic therapy for at least 6 months before the skin biopsy specimen and serum samples’ collection. There was no difference between patients and control subjects regarding age, smoking, medicaments or alcohol intake. The sera samples were collected at the Department of Laboratory Diagnostics, University Hospital Dubrava, Zagreb, Croatia) and used for the assessment of body redox homeostasis. Patient evaluation included history, physical examination, pathology findings and appropriate imaging techniques.

The study was approved by the University Hospital Dubrava Ethics Committee, and written informed consent was obtained from each person involved. All procedures were in accordance with recommendations in the Helsinki Declaration of 1975.

### 4.2. Skin Biopsy Specimen and Histopathologic Examination

Because the skin biopsy is the gold standard for the diagnosis of cutaneous vasculitis and also necessary for the detection of cutaneous vascular immune complexes by direct immunofluorescence (DIF) [4], it was performed in patients with suspected SVV. The 3–5 mm biopsy specimens were obtained under local anesthesia, from the fresh, most palpable purpuric vasculitic skin changes, not previously treated with local or systemic therapy. Special attention was given to the optimal time of taking skin biopsies. Skin biopsy was performed within 24–48 h after the appearance of vasculitic lesions because, similar to HE evaluation, the presence of diagnostic DIF patterns is inversely correlated with the lesion age [22]. The biopsy specimens from 30 control subjects were taken during the excision of benign, noninflammatory disorders, in which 3–5 mm of healthy skin was taken from the edge of the lesion.

The biopsy samples were placed in 10% buffered formalin and immediately processed in the histopathologic laboratory. Histologic sections (4 μm) of paraffin-embedded tissue were prepared. The sections were examined by a pathologist well experienced in the histopathology of vasculitis. All patients with SVV were divided into three groups on the basis of histological findings: (1) florid active inflammation (florid phase), a severe form with extensive fibrinoid necrosis, ulceration or glandular necrosis; (2) active inflammation (active phase), inflammatory infiltrate with leukocytoclasis, focal fibrinoid necrosis of blood vessel walls; and (3) resolution of inflammation (regressive phase), scarce infiltrate, rare foci of leukocytoclasis and without fibrinoid necrosis. 

### 4.3. Immunohistochemical Detection of Acrolein–Protein Conjugates

Skin samples of 97 subjects were analyzed for the presence of acrolein–protein conjugates similarly as described before [11]. Briefly, 4 μm sections made from paraffin blocks were deparaffinized, rehydrated and subsequently quenched for endogenous peroxidase activity with 3% hydrogen peroxide (H_2_O_2_, Kemika, Zagreb, Croatia). Immunohistochemical staining was performed using genuine monoclonal antibodies directed against acrolein–Lys conjugates and an EnVision detection kit (Dako, Glostrup, Denmark). 3,3′-Diaminobenzidine tetrahydrochloride (Dako) was used as a chromogen. The sections were counterstained with hematoxylin. Negative control slides, in which the test antibody was omitted and replaced by control diluents, were included in all experiments. Acrolein positivity was estimated using a semiquantitative method by an experienced pathologist (− = no positivity, + = weak focal positivity, ++ = moderate positivity, +++ = strong diffuse positive reaction). 

### 4.4. Measurement of Inflammatory and Immunological Parameters in the Prospective Cohort Samples

The inflammatory and immunological parameters were measured by standard laboratory methods in the context of routine diagnostic evaluation. The following inflammatory, immunological and biochemical parameters, obtained as part of the performed diagnostic processing, were included in the statistical analysis: CRP, erythrocytes (Er), Hgb, Htc, average volume of erythrocytes (Mean Corpuscular Volume (MCV)), average hemoglobin of erythrocyte (Mean Corpuscular Hemoglobin, MCH), average concentration of hemoglobin (Mean Corpuscular Hemoglobin Concentration, MCHC), red cell distribution width (RDW), Fe, UIBC, TIBC, ferritin, Trc, mean platelet volume (MPV), LEU, NEUT, MONO, lymphocytes, eosinophils, basophils, blood glucose (GUK), triglycerides (TRIG), cholesterol (CHOL), high-density lipoprotein cholesterol (HDL), low-density lipoprotein cholesterol (LDL), urea, CREAT, urate, potassium (K), sodium (Na), chlorides (Cl), calcium (Ca), PHOS, total bilirubin (BIL), aspartate aminotransferase (AST), alanine aminotransferase (ALT), GGT, alkaline phosphatase (ALP), LDH, CK, total proteins (PROT) and albumins (ALB).

### 4.5. Total Serum Peroxides 

Total serum peroxides (TSP) were measured using a commercially available enzymatic assay (Omnignostica GmbH & Co. KG, Höflein, Austria) and as described before [7]. Briefly, 10 µL of the serum samples was transferred into 96-well plates in duplicates and incubated with a mixture of horseradish peroxidase, tetramethylbenzidine and phosphate buffer for 30 min. The reaction was stopped by the addition of a stop solution and the absorbance measured at 450 nm using a plate reader (Easy-Reader 400 FW, SLT Lab Instruments GmbH, Salzburg, Austria). The H_2_O_2_ was used as a standard and the results are expressed as μM of H_2_O_2_ equivalents.

### 4.6. Total Antioxidant Capacity 

Commercially available enzymatic assay was used to measure total antioxidative capacity (TAC) (Omnignostica GmbH & Co. KG, Höflein, Austria). Briefly, 10 µL of the serum samples was incubated with a mixture containing H_2_O_2_ for 5 min, followed by the addition of peroxidase and TMB and incubation for 10 min at room temperature. The reaction was stopped by the addition of a stop solution, and the absorbance was measured at 450 nm using a plate reader (Easy-Reader 400 FW, SLT Lab Instruments GmbH, Salzburg, Austria). Hydrogen peroxide was used as a standard, and the results are expressed as μM H_2_O_2_ equivalents. Uric acid (2-,6-, 8-trihydroxypurine, Sigma, Taufkirchen, Germany) was used as a standard, and the results are expressed as mg/mL of uric acid equivalents.

### 4.7. Statistical Analysis

Descriptive statistics were shown as the mean ± SE. The significance of differences between groups was assessed using the Student’s *t*-test and Chi-square test. The correlation between different parameters was performed using Pearson correlation. Statistica 7.0 (StatSoft Inc., Tulsa, OK, USA), SigmaStat 2.0 (Jandel Scientific Corporation, San Raphael, CA, USA) and IBM SPSS Statistics 22 software for Microsoft Windows were used. Differences with p less than 0.05 were considered statistically significant. The Benjamini–Hochberg false discovery rate (FDR) method at *p* < 0.05 was used when comparing parameters between control and SVV patients’ samples. 

## Figures and Tables

**Table 1:** General characteristics of subjects whose skin biopsies were analyzed for the presence of acrolein–protein conjugates.

Parameters	SVV Patients	Control
Number of subjects	67	30
Male	33	12
Female	34	18
Age at diagnosis (years)	56.4 ± 16.5	52.1 ± 19.8
Age range (years)	19–88	20–85
**Disease activity**		
Florid phase	11	-
Active phase	45	-
Regressive phase	11	-

**Table 2:** Characteristics of the prospective patients with SVV (*n* = 30).

Parameters	*n*	%	Systemic Symptoms	*n*	%
Male	15	50.0%	**Temperature**		
Female	15	50.0%	Yes	15	50.0%
			No	15	50.0%
**Age at diagnosis (years)**					
Average	56.63		**Arthralgias**		
Range	19–79		Yes	11	36.7%
			No	19	63.3%
**Illness duration at diagnosis**					
Average	8.7 months		**Swelling of the joints**		
Range	1 day–6 years		Yes	11	36.7%
			No	19	63.3%
**Disease activity**					
Florid phase	5	16.7%	**Presence of blood in urine**		
Active phase	20	66.7%	Yes	9	30.0%
Regression period	5	16.7%	No	21	70.0%
**Associated chronic disease**			**Cough**		
Yes	24	80.0%	Yes	9	30.0%
No	6	20.0%	No	21	70.0%
**Associated malignancy**			**Headaches**		
Yes	1	3.3%	Yes	7	23.3%
No	29	96.7%	No	23	76.7%
**Smoking**			**Myalgias**		
Yes	7	23.3%	Yes	5	16.7%
No	23	76.7%	No	25	83.3%
**Alcohol intake**			**Abdominal pain**		
Yes	1	3.3%	Yes	3	10.0%
No	29	96.7%	No	27	90.0%
**Associated symptoms of systemic vasculitis**			**Diarrhea**		
Yes	28	93.3%	Yes	3	10.0%
No	2	6.7%	No	27	90.0%
**Lethal outcome**			**Paresthesias**		
Yes	1	3.3%	Yes	2	6.7%
No	29	96.7%	No	28	93.3%

## Data Availability

The data that support the findings of this study are available from the corresponding author upon reasonable request.

## Supplementary Materials

The following are available online, Table S1: Comparative analysis of inflammatory and biochemical parameters between SVV and healthy subjects from the prospective cohort.

## Sample Availability

MCEO is available from N.A.L.-D.
