# Supplementary material for: The Onset of Systemic Oxidative Stress Associated with the Accumulation of Lipid Peroxidation Product Acrolein in the Skin of Patients with Small-Vessel Vasculitis
Source: Molecules. 2021 Apr 17;26(8):2344. doi: 10.3390/molecules26082344 (PMC8073584; doi:10.3390/molecules26082344)
Supplement: Supplementary file 1 [file molecules-26-02344-s001.zip › molecules-1164261-supplementary.pdf]

Supplementary Table S1. Comparative analysis of inflammatory and biochemical parameters between SVV and healthy subjects from the prospective cohort

Parameter	Ref. interval	Healthy controls (n=30)	SVV subjects (n=30)			
			All SVV	Florid phase	Active phase	Regression phase
GUK	4.4-6.4 mmol/L	6.07 ± 0.24	6.17 ± 0.50	7.76 ± 1.28	5.56 ± 0.54 +	7.38 ± 1.84
UREA	2.8-8.3 mmol/L	6.86 ± 0.43	12.52 ± 1.71 * $	14.47 ± 2.60 *	13.11 ± 2.48 *	8.28 ± 2.61 +
CREAT	63-107 µmol/L	92.00 ± 3.55	210.04 ± 37.03 * $	164.43 ± 43.93 *	261.07 ± 57.88 *	100.40 ± 13.02 + #
URAT	134-337µmol/L	324.67 ± 17.62	390.22 ± 30.31 +	532.00 ± 87.40 * #	359.71 ± 27.01 +	319.75 ± 61.87 +
PROT	60-81 g/L	70.61 ± 2.44	68.26 ± 1.69	65.00 ± 3.70	67.93 ± 1.82	73.50 ± 5.81
ALB	41-52 g/L	42.63 ± 1.60	38.41 ± 1.50	38.30 ± 3.64	36.70 ± 1.32 *	48.00 ± 5.00
BIL	3-20µmol/L	16.86 ± 4.28	17.08 ± 5.04	15.45 ± 3.33	11.50 ± 2.11	46.37 ± 38.07
TRIG	<1.7 mmol/L	1.63 ± 0.33	2.32 ± 0.49	2.88 ± 0.87	2.38 ± 0.67	0.80 ± 0.11 +
CHOL	<5.0 mmol/L	4.84 ± 0.29	5.06 ± 0.34	5.68 ± 0.98	4.76 ± 0.40	5.64 ± 0.21
HDL	>1.0 mmol/L	1.38 ± 0.10	1.23 ± 0.11	1.40 ± 0.27	1.11 ± 0.10 *	1.88 ± 0.00
LDL	<3.0 mmol/L	2.79 ± 0.29	2.85 ± 0.28	3.28 ± 0.82	2.55 ± 0.21	3.55 ± 0.00
K	3.9-5.1mmol/L	4.35 ± 0.07	4.57 ± 0.13	4.59 ± 0.32	4.60 ± 0.18	4.74 ± 0.32
Na	137-146 mmol/L	141.56 ± 0.43	138.85 ± 0.94 * $	139.86 ± 2.12	138.67 ± 1.27	137.00 ± 1.48
Cl	97-108 mmol/L	105.05 ± 0.65	103.67 ± 0.96	104.75 ± 3.54	103.57 ± 1.06	102.67 ± 2.19
Ca	2.14-2.65 mmol/L	2.44 ± 0.02	2.35 ± 0.05	2.42 ± 0.11	2.28 ± 0.05	2.50 ± 0.12
PHOS	0.79-1.42 mmol/L	1.05 ± 0.03	1.36 ± 0.07	1.34 ± 0.15	1.38 ± 0.08	1.28 ± 0.17
AST	11-38 U/L	29.68 ± 3.71	41.96 ± 20.90	25.14 ± 5.62	18.80 ± 2.40	129.80 ± 108.06
ALT	12-48 U/L	28.65 ± 3.33	32.23 ± 7.52	26.00 ± 4.12	23.93 ± 4.41	60.60 ± 36.99
LDH	0-241 U/L	185.96 ± 6.09	221.04 ± 25.19	246.00 ± 55.36	190.93 ± 17.35	283.40 ± 97.21
CK	0-177 U/L	154.14 ± 40.52	52.06 ± 9.23 *	42.60 ± 6.01 *	51.27 ± 12.99 *	70.00 ± 30.01
GGT	9-35 U/L	43.43 ± 10.97	69.12 ± 16.56	53.83 ± 20.12	64.20 ± 20.10	110.50 ± 70.08

ALP	64-153 U/L	80.12 ± 7.43	88.96 ± 9.64	76.00 ± 9.38	89.47 ± 14.49	106.50 ± 23.74
Fe	8-30 µmol/L	17.01 ± 1.66	10.16 ± 1.92 * $	16.55 ± 4.48 #	8.18 ± 2.25 * +	10.30 ± 3.20 *
UIBC	26-59 µmol/L	42.83 ± 2.73	37.83 ± 3.60	27.15 ± 6.14 *	37.48 ± 3.00	61.30 ± 19.70
TIBC	49-75 µmol/L	59.77 ± 2.35	48.41 ± 3.35 * $	43.70 ± 2.93 *	46.11 ± 3.43 *	71.60 ± 16.50 +
CRP	< 5.0 mg/L	2.14 ± 0.24	45.88 ± 14.39 * + $	12.96 ± 8.14 * #	69.14 ± 21.48 * +	13.46 ± 7.94 * + #
Ferritin	20-200µg/L	108.27 ± 26.14	510.65 ± 128.37 *	917.75 ± 311.40 *	449.91 ± 139.91 *	30.50 ± 22.50 * + #
LEU	3.4-9.7x10^9/L	6.16 ± 0.25	10.34 ± 0.87 * $	12.93 ± 2.23 *	10.21 ± 0.84 *	8.46 ± 2.37
Er	4.34-5.72x10^{12}/L	4.43 ± 0.08	3.92 ± 0.14 * $	4.05 ± 0.31	3.66 ± 0.15	4.43 ± 0.32
Hgb	138-175 g/L	132.14 ± 2.69	115.73 ± 4.19 * $	123.29 ± 7.38	106.73 ± 5.06 *	130.00 ± 7.77 #
Htc	0.415-0.53L/L	4.68 ± 4.29	0.35 ± 0.01 *	0.37 ± 0.02 *	0.32 ± 0.01 *	0.40 ± 0.02 * #
MCV	83-97.2fL	89.71 ± 0.87	89.21 ± 0.88	92.70 ± 1.59	88.11 ± 1.03	90.14 ± 2.75
MCH	27.4-33.9 pg	29.82 ± 0.36	29.31 ± 0.28	30.33 ± 0.43	29.03 ± 0.37	29.52 ± 0.87
MCHC	320-345 g/L	332.27 ± 1.37	328.84 ± 1.69	327.17 ± 3.57	329.47 ± 2.37	328.80 ± 2.58
RDW	9-15%	13.62 ± 0.18	14.53 ± 0.41	14.10 ± 0.75	14.26 ± 0.44	15.23 ± 1.30
Trc	158-424 x10^9/L	218.97 ± 11.19	284.46 ± 25.61 * $	285.57 ± 53.99	290.80 ± 35.38 *	311.20 ± 68.11 *
MPV	6.8-10.4 fL	7.97 ± 0.17	8.01 ± 0.29	8.35 ± 0.91	8.17 ± 0.31	6.98 ± 0.15 *
NEUT	2.06-6.49 x10^9/L	3.77 ± 0.23	8.51 ± 0.84 * # $	11.00 ± 2.51 * #	77.81 ± 2.41 * +	73.95 ± 6.03 * +
LYMPH	1.19-3.35 x10^9/L	1.55 ± 0.09	1.53 ± 0.16 #	2.06 ± 0.58 #	13.99 ± 1.60 * +	16.98 ± 3.78 * +
MONO	0.12-0.84 x10^9/L	0.43 ± 0.02	0.59 ± 0.06 #	0.68 ± 0.21 #	5.93 ± 0.64 * +	5.75 ± 1.13 * +
Eos aps	0-0.43 x10^9/L	0.12 ± 0.02	0.10 ± 0.02 #	0.09 ± 0.03 #	0.99 ± 0.24 * +	1.18 ± 0.63 * +
Baso aps	0-0.06 x10^9/L	0.02 ± 0.01	0.02 ± 0.01 #	0.04 ± 0.02 #	0.19 ± 0.04 * +	0.48 ± 0.17 * +

* $p < 0.05$ in respect to control; + $p < 0.05$ in respect to the fluoride phase; # $p < 0.05$ in respect to active phase; $ Benjamini-Hochberg $p < 0.05$
